# Adult Patient Satisfaction with Nursing Care Services and Associated Factors Among Admitted Patients at Saint Paul’s Hospital, Millennium Medical College, Addis Ababa, Ethiopia, 2022: A Cross-Sectional Study

**DOI:** 10.31661/gmj.v12i.2906

**Published:** 2023-08-19

**Authors:** Bantalem Amanu Bogale, Sindew Mahmud Ahmed, Aynie Birhane Gebrekidan, Getachew Amanu Bogale

**Affiliations:** ^1^ Department of Nursing, Saint Paul’s Hospital Millennium Medical College, Addis Ababa, Ethiopia; ^2^ School of Nursing, Kotebe University of Education, Addis Ababa, Ethiopia; ^3^ Department of Nursing, Kea-Med College, Addis Ababa, Ethiopia; ^4^ Department of Public Health, Kutaber City Heath Office, Dessie, Ethiopia

**Keywords:** Patient Satisfaction, Adult, Inpatients, Nursing Care, Healthcare Service

## Abstract

Background: Providing comprehensive nursing care and ensuring patient satisfaction are essential health performance indicators worldwide. Despite some efforts to improve patient satisfaction with nursing care, the approach in developing countries, including Ethiopia, remains insufficient. This study aimed to assess the level of adult patient satisfaction and identify the factors affecting satisfaction. Materials and Methods: This cross-sectional study included 407 participants selected using a simple randomization technique. The samples were distributed using proportional allocation to each selected adult inpatient department. The participants were interviewed using a modified structured Amharic version of the Newcastle Satisfaction with Nursing Scale. Bivariate and multivariable logistic regression analyses were also performed.Results: The overall level of patient satisfaction with nursing care services was 54.3%. Respondents without formal education (P=0.010), male sex (P=0.041), free service consumers (P0.001), and health insurance users (P0.001) were significantly associated with satisfaction with nursing care. In addition, previously hospitalized patients (P=0.001), governmental workers (P0.001), and patients admitted to the medical ward (P=0.010) were associated with patient dissatisfaction with nursing care services.Conclusion: This study revealed that adult patient satisfaction with nursing care services is low. A previous admission history, higher education level, paying cash for services, and private and governmental workers were significant predisposing factors for dissatisfaction with nursing care. On the other hand, patients without formal education, free-service consumers, and male sex were significant predictors of satisfaction with nursing care services. Therefore, hospital administrators are encouraged to focus on patients’ needs and expectations.

## Introduction

Patient satisfaction is defined as people’s expectations of healthcare services based on their health, disease, quality of life, and other requirements [1]. The American Nurses Association defines patient satisfaction with nursing care as patients' perceptions of the care they receive from the nursing staff during hospitalization [2]. Many researchers describe it as an individual's assessment of how well the healthcare service provided meets their expectations, preferences, beliefs, and needs from their unique perspective, resulting in patient satisfaction [3].

Patient satisfaction is a key determinant of the quality of care delivery, and as an indicator accepted worldwide, it must continuously be part of institutional standards [4]. Thus, the healthcare sector has strived to transform and expand globally to meet patient needs, and quality healthcare is now seen as a right rather than a privilege [5]. The World Health Organization (WHO) and International Council of Nurses (ICN) have set the crucial goal of providing the best possible healthcare services for all people and maintaining high patient satisfaction [6][7].

Nurses are the first-line professionals for patient care and spend most of their time in the hospital with patients. Overall, nursing care plays a significant role in patient satisfaction during hospitalization [1]. The assessment of patient satisfaction level is important for identifying factors that influence patients' needs and quality of care [8]. Unfortunately, developing countries, including Ethiopia, have not conducted patient satisfaction surveys to monitor and assess the quality of care delivery compared to developed countries [9].

The Ethiopian Federal Ministry of Health (FMOH) has been working on reforms to improve the quality of nursing care for better patient satisfaction across the country over the past 10 years [10].

These include the launch of the Compassionate, Respectful, and Caring (CRC) project and introducing national standards for quality improvement in nursing care services; and audit tools. [10].

However, in Ethiopia, the quality of healthcare services is inadequate to improve and maintain patient satisfaction with nursing care. Furthermore, patient dissatisfaction can lead to poor adherence to treatment and poor health outcomes [11].

## Materials and Methods

Study Design and Setting

This descriptive cross-sectional study was conducted at the Saint Paul’s Hospital, Millennium Medical College, Addis Ababa, Ethiopia. According to the hospital's human resources report, Saint Paul’s Hospital Millennium Medical College is a 700-bed specialized hospital providing care to the underserved population on the outskirts of Addis Ababa. The hospital serves an average of 200,000 patients and clients daily and has a catchment population of over 5 million.

There are over 3,162 clinical and non-clinical staff in over 13 departments. Data collection started on July 1 and ended on July 30, 2022, G.C.

The study included patients who were admitted to Saint Paul's Hospital, Millennium Medical College's medical, surgical, ENT/maxillofacial, and obstetrics-gynecology departments; had a hospital stay of >2 days; were >18 years old; were conscious, coherent, and mentally stable; and were willing to participate in the study.

Study Sample and Sampling Procedure

The sample size (n) was calculated using the single population proportion formula (p) with an adult patient satisfaction with nursing care magnitude of 49.2% [12], a degree of precision (d) of 0.05, and a 95% confidence interval (Zα/2). After adding a 10% non-response rate, the final sample size was 422. The final sample size (422) was allocated proportionally to each selected department to select a representative sample of patients, considering the number of hospitalizations in each department over the last 12 months. Then, a simple random sampling method was employed to select eligible respondents for the face-to-face interviews. A lottery selection process was used to select participants who met the inclusion criteria from each department. This was achieved by asking the participants to choose from a roll of paper that had the words "Yes" and "No" written on it. Those who picked "Yes" were entered into the study, whereas those who picked "No" were excluded.This led to the inclusion of 268 patients from the Obstetrics and Gynecology Department, 101 patients from the surgical department, 33 patients from the ENT/maxillofacial department, and 20 patients from the internal medicine department.

Data Collection Tool

The modified Amharic version of the "Newcastle Satisfaction with Nursing Scale" (NSNS) tool, adapted from a similar study conducted in Ethiopia, was used to measure patients satisfaction with nursing care [13]. The questionnaire for the current study was divided into three sections: Part I, which provides a guide on socio-demographic data; Part II, which provides a guide on patient satisfaction with nursing care and includes 19 items on a 5-point Likert scale (1=not at all satisfied, 2=barely satisfied, 3=quite satisfied, 4=very satisfied, and 5=completely satisfied) [13][14]; and Part III, which provides a guide on organization and patient admission-related issues.

Data Collection Process and Personnel

Two diploma nurses and one BSc nurse participated actively in the data collection. Theinvestigators provided one day of training session on research instruments and data collection techniques to the data collectors. The investigators and supervisor collected the completed questionnaires daily and reviewed them for completeness and missing values. The quality of the data was checked before, during, and after collection. At Saint Peter's Hospital, 5% of the study participants were pretested before data collection, and questions that were difficult for them to understand were restated in a way that was clear to the participants. All study participants were assured of their confidentiality and anonymity. Information was collected through face-to-face interviews.

Operational Definition

Satisfaction was classified into two categories: satisfied and dissatisfied. On this basis, "Satisfied" refers to participants who scored greater than or equal to the mean value for NSNS satisfaction questions, and "Dissatisfied" refers to participants who scored below the mean value for NSNS satisfaction questions [13][14].

Statistical Analysis

Before exporting the data to IBM SPSS Statistics version 26 (SPSS Inc., Chicago, IL, USA) for analysis, the data were modified, coded, and entered into EpiData version 4.6 (EpiData Association, Odense, Denmark). Descriptive statistics were used to present the results of the data analysis.

Bivariate and multivariable logistic regression models were used to identify the association between adult patient satisfaction and its associated factors. Bivariate analysis was primarily used to fit the multiple logistic regression models, and variables with P<0.25 were selected and entered into the multivariable logistic regression model. Finally, variables with a statistically significant association with the outcome variable were identified using P<0.05, with a 95% confidence interval.

Ethics Approval and Consent to Participate

Ethical clearance was secured from the Institutional Review Board (IRB) of Saint Paul’s Hospital Millennium Medical College (approval identification PM23/54), and permission and support letters were secured from the Research and Ethical Review Board of Kea-Med College (OF02/KMC/5470/14) to the respective hospital administrators before data collection. Informed consent was obtained from the volunteer participants. Each respondent was informed by the data collectors of the purpose and expected benefits of the study. The respondents were also given the right to refuse to participate in the study and to withdraw at any time during the study. Confidence and anonymity were ensured throughout the study. In addition, the study was conducted in conformity with all applicable rules and regulations.

## Results

Socio-demographic Characteristics of Study Participants

A total of 407 patients participated in this study, with a response rate of 96.4%. Most of the study participants, 181 (44.5%), were aged between 18 and 30 years. More than three-fourths (311, 76.7%) of the participants were female. Regarding the educational status of patients, 262 (64%) had a college/university and secondary education, while the remaining 145 (36%) could not read and write and had only primary education. Regarding income level, 118 (29%) earned a monthly income of Birr >5000, while the remaining 113 (21.5) and 173 (42.5%) had a monthly income of Birr 1000-5000 and Birr <1000, respectively (Table-1).

Organization and Patient Admission Related Characteristics

Regarding the units of admission, 257 (63.1%) participants were from the obstetrics and gynecologic department, 99 (24.3%) were from the surgical department, and 19 (4.7%) were from the medical department, while the remaining 32 (7.9%) were from the ENT/maxillofacial department. In terms of previous admission history, nearly all 349 (85.7%) participants had no prior patient hospitalization, while the remaining 58 (14.3%) had at least one admission history (Table-2).

Level of Adult Patient Satisfaction with Inpatient Nursing Care

Patient satisfaction with inpatient nursing care was 54.3% (95% CI=49.6-59.2) with a mean score of 4.106 (SD± 0.81). The "nurse's manner of going about their work" (216, 53.1%) and "there always being a nurse around when needed" (216, 53.1%) were the highest parameters for nursing care satisfaction, followed by the "willingness of nurses to respond to patients' requests" (210, 51.6%). On the contrary, "Nurse’s awareness of patients' needs" (127, 31.2%), and "how nurses helped put patients' relatives’ or friends' minds at rest" (129, 31.7%) were found to be the lowest parameters for patient satisfaction (Table-3).

Factors Associated with Patient Satisfaction in Nursing Care

In the bivariate analysis, 15 predictor variables were used, and those with a P-value of less than 0.25 were entered into the multivariate logistic regression model. The outcome variable was significantly associated with only six predictor variables. The variables that had a significant association with adult patient satisfaction with nursing care services were the respondents' gender, educational level, occupation, admission ward, prior hospitalization, and method of service charge. Respondents without formal education (Adjusted odds ratio (AOR)=8.482; 95% CI=1.678-42.873) (P=0.01), male sex (AOR=2.487; 95% CI=1.038-5.959) (P=0.041), patients who were free service consumers (AOR=6.650; 95% CI=2.677-16.517) (P<0.001), and those using health insurance (AOR=7.309; 95% CI=3.122-17.110) (P<0.001) were associated with patient satisfaction with nursing care services. Patients with a previous admission history (AOR=0.261; 95% CI=0.122-0.560) (P=0.001), as well as governmental workers (AOR=0.090; 95% CI=0.026-0.310) (P<0.001), private workers (AOR=0.148; 95% CI=0.048-0.453) (P=0.001), and patients admitted to the medical ward (AOR=0.160; 95% CI=0.039-0.649) (P=0.010) were associated with patient dissatisfaction with nursing care services (Table-4).

## Discussion

**Table:** Table1. Socio-demographic Characteristics of the Respondents at SPHMMC (n=407) of Addis Ababa, Ethiopia, 2022

Variable	Category	Frequency	Percent %
Sex	Male	96	23.6
	Female	311	76.4
Age	18-30	181	44.5
	31-40	156	38.3
	41-50	19	4.7
	51-60	30	7.4
	>60	21	5.2
Level of education	Unable to read and write	12	2.9
	Able to read and write	22	5.4
	Primary education	111	27.3
	Secondary education	95	23.3
	College/ University	167	41.0
Marital status	Single	62	15.2
	Married	327	80.3
	Divorced	12	2.9
	Widowed	6	1.5
Occupation	Private work/NGO	148	36.3
	Governmental	81	19.9
	Housewife	138	33.9
	Farmer	40	9.8
Income: birr/month	<1000 ETB	173	42.5
	1000-2500 ETB	47	11.5
	2501-5000 ETB	66	16.2
	>5000 ETB	118	29.0
Place of residency	Urban	330	81.0
	Rural	77	19.0

**ETB:** Ethiopian birr;**SPHMMC:** Saint Paul’s Hospital Millennium Medical College

**Table:** Table2. Organization and Patient Admission Related Characteristics of the Study Participants at SPHMMC (n=407) of Addis Ababa, Ethiopia, 2022

Variable	Category	Frequency	Percent	Satisfaction Level	
				Satisfied	Dissatisfied
Departments	Medical	19	4.7	11(57.9%)	8(42.1%)
	Surgical	99	24.3	33(33.3%)	66(66.7%)
	Gynecology-obstetrics	257	63.1	162(63.0%)	95(67.0%)
	ENT/Maxillofacial	32	7.9	15(46.9%)	17(53.1%)
Previous admission history	Yes	58	14.3	23(39.65%)	35(60.34%)
	No	349	85.7	198(56.7%)	151(43.3%)
Length of stay	2-7 days	218	53.6	81(37.2%)	137(62.8%)
	8-15 days	106	26.0	62(58.5%)	44(41.5%)
	16-30 days	59	14.5	37(62.7%)	22(37.3%)
	31-60 days	23	5.7	17(73.9%)	6(26.1%)
	>60 days	23	5.7	6(26.0%)	17(74.0%)
Comorbidity	Yes	20	4.9	8 (44.0%)	12(66.0%)
	No	379	93.1	208(54.9%)	171(45.1%)
	I do not know	8	2.0	5(62.5%)	3(37.5%)
Availability of assigned nurse	Yes	378	92.9	203(53.7%)	175(46.3%)
	No	4	1.0	2(50.0%)	2(50.0%)
	I do not know	25	6.1	16(64.0%)	9(36.0%)
Involving with treatment and care decision making	Yes	389	95.6	221(56.8%)	168(43.2%)
	No	18	4.4	0(0%)	18(100%)
Method of service charge	Free	214	52.6	135(63.0%)	79(37.0%)
	Health insurance	115	28.3	67(58.3%)	48(41.7%)
	Pay with cash	78	19.2	19(24.4%)	59(75.6%)
Number of beds per room	Single bed	2	0.5	0(0%)	2(100%)
	Two beds	4	1.0	2(50.0%)	2(50.0%)
	More than two bed	401	98.5	219(54.6%)	182(45.4%)

**Table:** Table3. Frequency of Adult Patient Satisfaction with Nursing Care (N=407)

Items	Satisfied N (%)	Dissatisfied N (%)
The amount of time nurses spent	172 (42.3%)	235(57.7%)
How capable nurses were at their job	191(46.9%)	216(53.1%)
There always being a nurse around when needed	216(53.1%)	191(46.9%)
The amount nurses knew about patients care	202(49.6%)	205(50.4%)
How quickly nurses came when patients called for them	204(50.1%)	203(49.9%)
The way the nurses made patients feel at home	181(44.5%)	226(55.5%)
The amount of information nurses gave to patients about their condition and treatment	165(40.5%)	242(59.5%)
How often nurses checked to see if patients were okay	182(44.7%)	225(55.3%)
Nurses' helpfulness	210(51.6%)	197(48.4%)
The way nurses explained things to patients	199(48.9%)	208(51.1%)
How nurses helped put patients' relatives' or friends' minds at rest	129(31.7%)	278(68.3%)
Nurses' manner in going about their work	216(53.1%)	191(46.9%)
The type of information nurses gave to patients about their condition and treatment	195(47.9%)	212(52.1%)
Nurses' treatment of patients as an individual	202(49.6%)	205(50.4%)
Willingness of nurses to listened patients worries and concerns	162(39.8%)	245(60.2%)
The amount of freedom patients was given on the ward	202(49.6%)	205(50.4%)
Willingness of nurses were to respond to patients' requests	210(51.6%)	197(48.4%)
The amount of privacy nurses gave to patients	207(50.9%)	200(49.1%)
Nurses' awareness of patients’ needs	127(31.2%)	280(68.8%)

**Table:** Table4. Association between Patient Satisfaction and Different Determinant Factors at SPHMMC, Addis Ababa 2022

Variables	Satisfaction Level		COR (95% CI)	AOR (95% CI)
	Satisfied	Dissatisfied		
Sex				
Male	44(45.83%)	52(54.17%)	1.561(0.986-2.473)	2.487(1.038-5.959) *
Female	177 (56.9%)	134 (43.1%)	1.00	1.00
Level of education				
Unable to read and write	10(83.3%)	2(16.7%)	7.846(1.666-36.957)	1.729(0.296-10.087)
Able to read and write	19(86.4%)	3(13.6%)	9.938(2.828-34.924)	8.482(1.678-42.873) *
Primary education	53(47.7%)	58(52.3%)	1.434(0.883-2.330)	1.051(0.566-1.951)
Secondary education	74(77.9%)	21(22.1%)	5.530(3.109-9.835)	6.409(0.103-13.237)
College/ University	65(38.9%)	102(61.1%)	1.00	1.00
Place of residency				
Urban	156(47.3%)	174(52.7%)	1.405(0.846-2.331)	0.929(0.453-1.905)
Rural	30 (39%)	47 (61%)	1.00	1.00
Occupation				
Private work/NGO	69(46.6%)	79(53.4%)	0.254(0.113-0.570)	0.148(0.048-0.453) **
Governmental	28(34.6%)	53(65.4%)	0.153(0.064-0.367)	0.090(0.026-0.310) ***
Housewife	93(67.4%)	45(32.6%)	0.600(0.263-1.366)	0.176(0.053-3.585)
Farmer	31(77.5%)	9(22.5%)	1.00	1.00
Departments				
Medical	11(57.9%)	8(42.1%)	0.364(0.134-0.990)	0.160(0.039-0.649) **
Surgical	33(33.4%)	66(66.6%)	1.240(0.482-3.192)	0.854(0.223-3.2690)
Gynecology/obstetrics	162(63.0%)	95(37.0%)	0.642(0.204-2.017)	0.259(0.048-1.389)
ENT/Maxillofacial	15(46.9%)	17(53.1%)	1.00	1.00
Admission history				
Yes	23(39.7%)	35(60.3%)	0.501(0.284-0.884)	0.261(0.122-0.560) **
No	198(56.7%)	151(43.3%)	1.00	1.00
Method of service charge				
Free	135(63.1%)	79(36.9%)	5.306(2.951-9.543)	6.650(2.677-16.517) ***
Health insurance	67(58.3%)	48(41.7%)	4.334(2.294-8.188)	7.309(3.122-17.110) ***
Pay with cash	19(24.4%)	59(75.6%)	1.00	1.00

**NB:** Variables having a P≤0.25 in bivariate analysis included in the multivariable analysis; *: Statistically significant at P<0.05, **: significant at P<0.01, ***: significant at P<0.001, 1.00 Reference, **AOR:** Adjusted odds ratio; **CI:** Confidence interval; **COR:** Crude odds ratio; **ENT:** Ear, nose, and throat

Nursing care is a key element of healthcare services. Thus, patients have the right to expect and receive high-quality nursing care service. Nursing staff are the most professional group and have the most contact with physicians and other health care professionals. As a result, nurses are more likely to influence patients' attitudes and behaviors toward receiving care, rehabilitation, and recovery processes [15][16].

This study revealed that overall satisfaction with nursing care was 54.3% (95% CI=49.6-59.2). Thus, the finding of this study were higher than a study done in Nepal (39%) [17]. This difference could be due to the variation in the level of nursing care services needed and expectations. However, the findings of this study are less significant than those conducted in England (60%) [18] and Pawie General Hospital in West Ethiopia (68.8%) [19]. This discrepancy may result from the subjective character of satisfaction and/or study, which might be due to the difference in operationalization and techniques used to measure satisfaction. Additionally, the differences might be influenced by the services that hospitals provide, community expectations for healthcare services, and cultural views and values.

Patients’ educational status was a significant predictor of patient satisfaction with nursing care services. Patients with no formal education were eight times (AOR = 8.482; 95% CI = 1.678-42.873) more likely to be satisfied than patients with a higher educational status. This finding is supported by other studies conducted in Ghana [20] and Debre Berhan, Amhara Region, Ethiopia [12]. A possible explanation is that patients without formal education had lower expectations, were less aware of healthcare services, and had less knowledge of nursing care than the educated patients. Structured education enables patients to compare their needs and expectations of care and services with the services they receive.

The present study also revealed that male patients were two times (AOR=2.487; 95% CI =1.038-5.959, P-value=0.041) more likely to be satisfied than female patients, which was similar to a study conducted in Ethiopia at Butajira General Hospital [21]. The possible justification could be the similarities in the operationalization of satisfaction, techniques used to determine satisfaction status, and the satisfaction scale tool. In contrast, a study conducted in Ethiopia at public hospitals in Addis Ababa reported higher satisfaction among female patients than among male patients [8]. Explanations for this difference include cultural and geographical factors. Additionally, compared with male wards, female wards reported increased patient turnover during the study. It affects nursing care services and leads to burnout and exhaustion in the provision of nursing care.

People using health insurance were (AOR=5.621; 95% CI=1.489-11.213) (P<0.001) more likely to be satisfied with nursing care than those who paid cash for services. This finding is supported by studies conducted in the Eastern Amhara Region, northeastern Ethiopia, and China [13][22]. This could be due to the detrimental effect of high medical expenses on patient satisfaction with nursing care and other healthcare services.

In terms of occupation, 91% of governmental workers (AOR=0.090; 95% CI=0.026-0.310) (P<0.001) and 85% of private employees (AOR=0.148; 95% CI=0.048-0.453) (P=0.001) were less likely to be satisfied than their farmer counterparts. This finding is consistent with that of a study conducted in public hospitals in the Amhara region of Northwest Ethiopia [23]. A possible explanation is that most government employees’ education is higher than their counterparts’, so structured education could influence the expectations of their rights, needs, and nursing care. In addition, most private employees were not free service consumers or health insurance users, therefore, they might be dissatisfied because of the unequal service they receive.

Hospitalization was also significantly associated with satisfaction with nursing care services, with those who had a hospitalization history of 74% (AOR=0.261; 95% CI:0.122-0.560) (P=0.001) less likely to be satisfied than their counterparts. This result is consistent with the findings of a meta-analysis conducted in Addis Ababa, Ethiopia [24]. This could be due to antecedent dissatisfaction and stressful events, including organization-related factors, therapeutic procedures, the service fees they incur, and the inadequate quality of services they receive. There was a significant relationship between admitting department and patient satisfaction; 84% of those admitted to the medical ward (AOR=0.160; 95% CI=0.039-0.649) (P=0.010) were more likely to be dissatisfied with nursing care than their counterparts. This finding was consistent with that of a study conducted in Ethiopia at public hospitals in Addis Ababa [25]. A possible explanation is that patients admitted to the medical ward typically have more serious conditions with worse prognosis and are confronted with stressful and uncomfortable situations. Additionally, the majority of patients admitted to the medical ward had experienced previous admissions and traumatic events such as therapeutic procedures and expensive service charges.

According to the findings of this study, patient satisfaction with inpatient nursing care is low. Educational status, previous hospitalization, sex, admitting department, and method of service charge were predictors of admitted adult patients' satisfaction with nursing care services. Among the NSNS satisfaction items, "the nurses' awareness of patients’ needs" (127, 31.2%), "nurse’s help for patients’ relatives or friends’ minds at rest" (129, 31.7%), "the nurses' willingness to listen to patients' worries and concerns" (162, 39.8%), "nurse’s awareness of patients’ needs" (43, 17.6%), and the amount of information nurses provided to patients about their condition and treatment were found to be the lowest parameters regarding patient satisfaction.

## Conclusion

Approximately half of the study participants were dissatisfied with nursing care among patients admitted to Saint Paul’s Hospital, Millennium Medical College, Addis Ababa, in the surgical, medical, obstetric-gynecological, and ENT/maxillofacial departments. Compared with many published national and international studies, the current study revealed that admitted adult patients had a low level of satisfaction with nursing care services. Among the educational level, being male, not having formal education, and health insurance usage were found to be significant factors in patient satisfaction with nursing care. On the other hand, admission ward (medical), being a government worker, and having a history of admission were found to be significant factors of patient dissatisfaction with nursing care. Therefore, hospital administration should emphasize the needs and expectations of patients.

## Acknowledgments

We are incredibly grateful to all data collectors and study participants for their voluntary participation in the interview process. We also extend our acknowledgment to Saint Paul’s Hospital millennium Medical College administrators who gave their time and information to make this study possible. Additionally, we would like to express our sincere thanks to the University of Newcastle's NSNS team for enabling us to use their NSNS questionnaire in this research. Finally, we would like to thank Research Square for posting our paper as a preprint and allowing us to improve our paper with feedback from the community prior to peer review in the journal.

## Conflict of Interest

The authors declare that they have no competing interests.
